# Introducing *Alzheimer's & Dementia: Translational Research & Clinical Interventions*, an open access journal of the Alzheimer's Association

**DOI:** 10.1016/j.trci.2015.06.002

**Published:** 2015-07-01

**Authors:** Lon S. Schneider

**Affiliations:** Keck School of Medicine of the University of Southern California, Los Angeles, CA, USA

*Alzheimer's & Dementia: Translational Research & Clinical Interventions* is an open access journal of the Alzheimer's Association. The journal's mission is to advance and expedite clinical translational research in Alzheimer disease and age-related cognitive impairment into improved care and therapies, while promoting high-visibility, open access communication across preclinical, clinical, and effectiveness research areas.

The journal will promote the dissemination of knowledge and research outcomes that inform translational research, early and late-stage clinical trials and development, clinical research bioinformatics, and clinical practice by providing an open access vehicle for communication among preclinical, translational, and clinical trials researchers.

By translational research we mean research that plans to involve or includes humans and therapeutic interventions that range from preclinical to late stage trials and health technology assessment. The interventions may include drugs, biologicals, devices, and the full range of psychotherapeutic, psychosocial, and other non-pharmacological approaches, as well as technology assessment and effectiveness, services research, and clinical application. Thus, *Alzheimer's & Dementia: Translational Research & Clinical Interventions* covers therapeutics, clinical development, methods, clinical research informatics, and trials for Alzheimer disease, age-related cognitive impairment, and other conditions associated with cognitive impairment in late-life.

## 1 Publishing in open access journals

There are several advantages of an online, open access journal when compared with subscription-based, print journals. These include rapid review, publication within a few weeks of final acceptance, and the ability to post full-color, high-resolution graphics, video clips, and substantial supplemental material, such as protocols, methods, data forms, and programing code. Open access authors hold the copyright to their article, and publication of their articles meet the National Institutes of Health's and other funders' requirements for rapid dissemination of research outcomes.

Open access journals hosted by major publishers charge publishing fees in lieu of charging for subscriptions. Invited commentaries, perspectives, reviews, correspondences, communications, and images are not charged. Fees are substantially reduced for members of the Alzheimer Association's International Society to Advance Alzheimer's Research and Treatment (ISTAART). Many organizations and funding agencies will pay the fee for authors [1], [2]. Details about open access publishing for Alzheimer-related research were discussed in our sister journal, *Alzheimer's & Dementia: Diagnosis, Assessment, & Disease Monitoring* and elsewhere [3], [4].

## 2 The Journal's title and content

The content for *Alzheimer's & Dementia: Translational Research & Clinical Interventions* is guided and informed by the *Common Alzheimer's Disease Research Ontology* [5], Categories C and E, in particular; and the *International Alzheimer's Disease Research Portfolio* that aims to capture global Alzheimer research funding as well [6]. These categories include topics focused on the identification and development of therapies for Alzheimer's disease from early discovery through late stage preclinical development, all stages of clinical testing, interventions, and trials, as well as non-pharmacological primary, secondary, and tertiary interventions, including cognitive training, behavioral, neuropsychological interventions, outcomes, and treatment development (Box 1).

Box 1 **Common Alzheimer's Disease Research Ontology**

Category C. Translational research and clinical interventions

1. Drug discovery
2. Preclinical therapy development
  Pharmacological and non-pharmacological interventions
3. Clinical trial design and methods
4. Proof of concept and feasibility trials
5. Prevention trials
6. Treatment trials – cognition
  Various interventions
7. Treatment trials – neuropsychiatric, behavioral and psychological comorbidity
  Pharmacological and non-pharmacological

Category E. Care, support, and health economics

1. Care interventions and quality of life
2. Technology assisted care
3. Caregiver support
4. Cultural values and beliefs
5. Economic burden

(From: Refolo LM, et al. Alzheimer's & Dementia. 2012;8:372-5 [1])

## 3 Publishing in *Alzheimer's & Dementia: Translational Research & Clinical Interventions*

*Alzheimer's & Dementia: Translational Research & Clinical Interventions* encourages the submission of studies describing preclinical, translational research with potential for clinical application, research from early human experimentation (experimental medicine), clinical trials, prevention studies, clinical research bioinformatics, systematic reviews, metaanalyses, commentary, and perspectives that have the potential to advance treatment of Alzheimer's disease pathology, neurodegeneration, and cognitive impairment, and improve patients' lives. This is research that bridges the laboratory and clinical settings, including molecular characterization, discovery, formulation, prevention, detection, diagnosis and treatment, with the overall goal of improving the clinical care.

The journal will publish laboratory studies of novel therapeutic interventions and clinical trials which evaluate new pharmacological, non-pharmacological, psychosocial, and environmental treatment paradigms from prevention to disease management. Studies describing public health and health economics research with potential application to the clinic or disease prevention are also encouraged.

The areas of clinical research informatics, i.e. informatics applied to clinical research contexts, that the journal promotes include (1) efficient and effective data collection and acquisition for clinical trials; (2) protocol designs based on prior clinical trials data; (3) patient recruitment; and (4) adverse events, monitoring, regulatory approaches, including clinical research data storage, processing and analysis, tools and technologies that facilitate clinical research, metrics, statistics, outcomes and trials management, and the informing of clinical research through analysis of large datasets.

*Alzheimer's & Dementia: Translational Research & Clinical Interventions* intends to provide a communication vehicle for reports on the “nuts and bolts” of clinical research: methods advancements, procedures, protocols, protocols design, implementation, outcomes, metrics, regulatory science, statistical modelling, and clinical research bioinformatics.

The editors and an international editorial board of clinician-scientists, scientists, and others will hold *Alzheimer's & Dementia: Translational Research & Clinical Interventions* to the same standards of the parent journal, *Alzheimer's & Dementia*. This online journal serves as an alternative to the parent journal in order to cover the translational research areas that the *Alzheimer's & Dementia* cannot accomplish because of its mission and limited space. Editorial and peer review is as rigorous for *Alzheimer's & Dementia: Translational Research & Clinical Interventions* as it is for its parent journal.

### 3.1 Types of articles

The journal invites the submission of original articles and commentaries. These include commentaries, perspectives, personal views, position papers, correspondence, images, graphs, videos, narrative, full-length, or mini-reviews, systematic reviews and metaanalyses, and full-length and brief research articles.

Examples of research articles include, but are not limited to, preclinical drug development, clinical trials reports, phase 1 to 3 clinical trials, such as dose-finding, safety, proof of concept, and efficacy trials, clinical experimental methods, designs (including protocols), outcomes development, statistical methods, pooled analyses, simulations, and modeling.

Narrative reviews should have a clearly stated topic, may be invited by the editors or submitted without invitation. Invited reviews will be peer-reviewed and edited. Uninvited reviews will be evaluated by the editors for peer-review. Systematic reviews and metaanalyses are considered as research articles. They may be full-length or brief, mini-reviews. The key points are that they are preplanned, protocol-driven, and address a hypothesis or question.

Commentaries may comment on current articles, policy, clinical research, funding, regulatory issues, and similar. They are opportunities to present particular views on a translational research or clinical trials topic in order to promote innovation.

Perspectives provide an opportunity to present particular views on a topic, and may address any issue within the translational research scope of the journal. Importantly, a perspective article should discuss implications and next steps specifically, and should not leave the reader hanging. Correspondence is welcomed, and may address earlier publications or general topics relevant to the journal.

All original articles should have a structured abstract and a “Research in Context” section. The latter is essentially a post script paragraph of 100 to 150 words that contains the essential point of the article and requires authors to be specific in defining future research directions or crucial questions.

## 4 The editorial team

The Editor-in-Chief of *Alzheimer's & Dementia: Translational Research & Clinical Interventions*, is Dr. Lon S. Schneider. The Senior Associate Editors are Drs. Steven T. DeKosky, Hiroko Dodge, Miia Kivipelto, Simon Lovestone, Rupert McShane, and Mary Sano. Each has made important contributions to translational research, and will provide breadth, depth, experience, and perspective on the journal's direction.

The participation of a broadly-skilled, large editorial board representing academics, research, industry, and charity is another extremely valuable contribution to the start of this new venture (http://www.trci.alzdem.com/content/edboard). Dr. Zaven S. Khachaturian, Editor-in-Chief of *Alzheimer's & Dementia*, has provided immeasurable and wise guidance and support. Dr. Ara S. Khachaturian, Executive Editor for *Alzheimer's & Dementia*, Dr. Peter J. Snyder, *Editor-in-Chief of Alzheimer's & Dementia: Diagnosis, Assessment, & Disease Monitoring*, (http://www.dadm.alzdem.com), and Dr. Kathleen M. Hayden, Senior Associate Editor for *Alzheimer's & Dementia* and Executive Editor of the two *Alzheimer's & Dementia* open access journals provide substantial support.

Mr. Terry Materese, Executive Publisher at Elsevier leads the publishing effort; and the Alzheimer's Association liaises with the *Alzheimer's & Dementia* executive editor through Drs. Heather Snyder, and Dr. Maria Carrillo, Vice President of Medical and Scientific Relations for the Alzheimer's Association.

The editors of *Alzheimer's & Dementia: Translational Research & Clinical Interventions* hope that the journal will be widely visited, read, and valued by people interested in late-life cognitive impairment and that the journal is seen as an important resource for the field.
